# Natural killer cells facilitate PRAME-specific T-cell reactivity against neuroblastoma

**DOI:** 10.18632/oncotarget.5657

**Published:** 2015-10-06

**Authors:** Lotte Spel, Jaap-Jan Boelens, Dirk M. van der Steen, Nina J.G. Blokland, Max M. van Noesel, Jan J. Molenaar, Mirjam H.M. Heemskerk, Marianne Boes, Stefan Nierkens

**Affiliations:** ^1^ Laboratory of Translational Immunology, University Medical Center Utrecht, Utrecht, The Netherlands; ^2^ Pediatric Blood and Marrow Transplantation Program, University Medical Center Utrecht, Utrecht, The Netherlands; ^3^ Department of Hematology, Leiden University Medical Center, Leiden, The Netherlands; ^4^ Princess Maxima Center for pediatric oncology, Utrecht, The Netherlands; ^5^ Department of Oncogenomics, Academic Medical Center, University of Amsterdam, Amsterdam, The Netherlands; ^6^ Department of Pediatric Immunology, University Medical Center Utrecht, Utrecht, The Netherlands

**Keywords:** class I MHC, immune evasion, PRAME, neuroblastoma, immunotherapy

## Abstract

Neuroblastoma is the most common solid tumor in children with an estimated 5-year progression free survival of 20–40% in stage 4 disease. Neuroblastoma actively avoids recognition by natural killer (NK) cells and cytotoxic T lymphocytes (CTLs). Although immunotherapy has gained traction for neuroblastoma treatment, these immune escape mechanisms restrain clinical results. Therefore, we aimed to improve neuroblastoma immunogenicity to further the development of antigen-specific immunotherapy against neuroblastoma. We found that neuroblastoma cells significantly increase surface expression of MHC I upon exposure to active NK cells which thereby readily sensitize neuroblastoma cells for recognition by CTLs. We show that oncoprotein PRAME serves as an immunodominant antigen for neuroblastoma as NK-modulated neuroblastoma cells are recognized by PRAME_SLLQHLIGL_/A2-specific CTL clones. Furthermore, NK cells induce MHC I upregulation in neuroblastoma through contact-dependent secretion of IFNγ. Our results demonstrate remarkable plasticity in the peptide/MHC I surface expression of neuroblastoma cells, which is reversed when neuroblastoma cells experience innate immune attack by sensitized NK cells. These findings support the exploration of NK cells as adjuvant therapy to enforce neuroblastoma-specific CTL responses.

## INTRODUCTION

Tumor cells evade immune surveillance most notably through changes in surface expression of membrane receptors, antigen presentation and/or initiation of an inhibitory microenvironment [1]. Cancer immunotherapies that target these immune evasion mechanisms have resulted in clinical benefits [2–4], providing experimental support for plasticity in the immunogenicity of the tumor and its microenvironment. Neuroblastoma, which is considered a poorly immunogenic tumor, accounts for 15% of childhood cancer deaths [5]. It is the most common solid tumor in infancy that manifests in extracranial neural tissues. Despite intense treatments, stage 4 neuroblastoma patients have merely 20% survival rates due to tumor relapses.

Recently, interest for immunotherapy in neuroblastoma patients has grown. Antibody therapy in neuroblastoma initially yielded promising event-free survival benefits [6], however tumor relapse still occurred in the majority of the patients. The cytotoxic effects of antibody therapy do not involve adaptive immune cells that exert specific immunity against the tumor antigen. To date, the best clinical results for anti-tumor immunotherapy were obtained with strategies that are aimed at generating or improving adaptive immune responses [7–10], such as adoptive T-cell therapy and checkpoint inhibitors. We therefore hypothesize that stimulating antigen-specific immune responses may be imperative for effective immunotherapy in neuroblastoma patients.

Antigen-specific immunotherapy requires the expression of tumor-associated antigen(s) (TAA) by the neuroblastoma cells and sufficient TAA-derived peptide/MHC I complex display on the neuroblastoma cell surface to allow for recognition by antigen-specific T-cells. Neuroblastoma has established several mechanisms for the direct evasion of CTL recognition. Most importantly, neuroblastoma tumors show down-regulated surface expression of major histocompatibility complex I (MHC I) molecules [11–14]. In addition, the expression of co-stimulatory and adhesion molecules which assist in CTL activation are also decreased on neuroblastoma surfaces [15–17]. Of note, the absence of MHC I on neuroblastoma tumors should trigger the active recognition by cytotoxic immune cells of the non-specific innate immune system: the natural killer (NK) cells. Neuroblastoma, however, adapted its surface display of activating and inhibitory NK cell receptors in order to also avoid NK-mediated killing [16–19].

Neuroblastoma further enforces its non-immunogenicity through the expression of only few TAA [20, 21], which limits the repertoire of antigen-specific CTLs responsive against neuroblastoma. TAA encoded by cancer/germline genes, including PRAME, NY-ESO1, MAGE and GAGE, can be expressed by neuroblastoma tumors [20, 21]. Whereas the expression of most cancer/germline antigens in neuroblastoma was shown to be very heterogeneous, PRAME expression was observed in 94% of stage 4 neuroblastoma samples. However, specific CTL activity against PRAME-expressing neuroblastoma cells has not yet been described.

Here we investigated whether neuroblastoma cells can be triggered into peptide/MHC I display, as a possible approach towards antigen-specific immunotherapy. Specifically, we investigated whether neuroblastoma cells that have experienced innate immune pressure by NK cells change their immunogenic potential towards adaptive CTLs. We found that neuroblastoma cells increase their surface levels of MHC I in response to active NK cells. This effect is contact-dependent and identifies interferon-gamma (IFNγ) as the major factor inducing MHC I upregulation, which could be recapitulated in patient-derived stage 4 neuroblastoma cell cultures. Furthermore, we show for the first time that PRAME serves as an immunogenic tumor antigen for neuroblastoma as NK-modulated neuroblastoma cells present PRAME-derived peptide/MHC I complexes and are now recognized by PRAME-specific CTLs. Our data reveals that the immunogenic potential of neuroblastoma is amendable to environmental signals, as opposed to being caused by irreversible genetic mutations, a feature that could contribute to the development of antigen-specific immunotherapies for neuroblastoma.

## RESULTS

### NK cell exposure upregulates surface MHC I on neuroblastoma

By virtue of actively suppressed MHC I membrane expression [11–14], neuroblastoma is a potential target for NK cells [22]. We investigated changes in neuroblastoma MHC I expression after NK cell immune pressure, considering that tumor cells often have the ability to escape immune-mediated cytotoxicity. As naive NK cells were shown unable to eradicate neuroblastoma cells, we additionally used NK cells that were prior stimulated with IL-2 and IL-15 (Fig. S1), similar to NK cells used in cellular immunotherapy.

**Figure 1:**
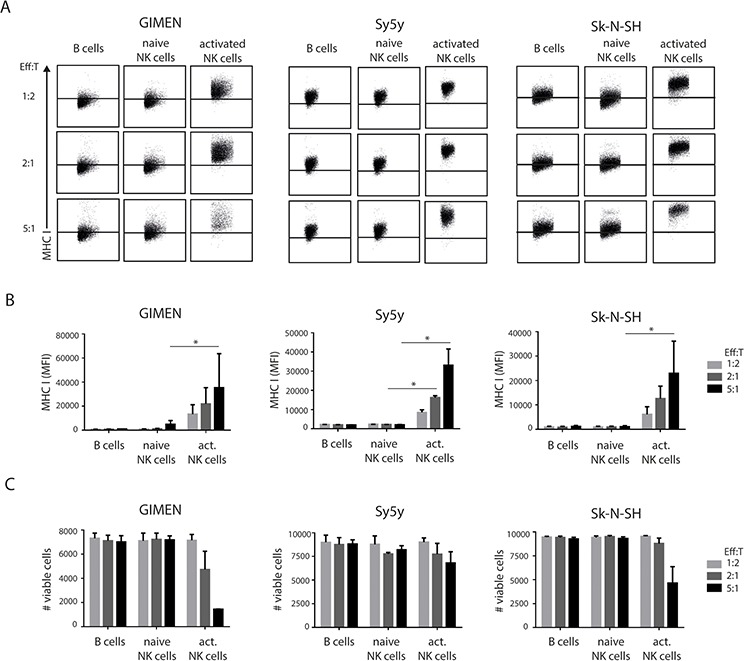
Class I MHC modulation on neuroblastoma cells by NK cell exposure **A.** 30,000 GIMEN, Sy5y or Sk-N-SH cells were co-cultured with B cells, naive NK cells or activated NK cells at indicated effector:target ratios for 24 hours. Neuroblastoma cells were harvested and stained for MHC I. MHC I levels were measured by flow cytometry. Representative FACS plots are shown. Data was quantified in **B.** (*n* = 3). **C.** Viable neuroblastoma cells were counted for each condition.

NK cells were isolated from healthy donor PBMCs and added to established neuroblastoma cell cultures, in increasing effector:target ratios (co-cultures of 15,000 or 60,000 or 150,000 NK cells with 30,000 neuroblastoma cells; O/N, 37°C). B cells purified from the same donor PBMCs served as a control population. We observed the significant upregulation of MHC I at the cell surface of GIMEN, Sy5y and Sk-N-SH neuroblastoma lines (Fig. 1AB), specifically when exposed to activated NK cells. With increasing amounts of NK cells, the average increase in MHC I expression was 8-, 15- and 26-fold. Stimulation of neuroblastoma cells with the IL2/IL15 cytokine mix alone did not induce MHC I upregulation (Fig. S2). Furthermore, activated NK cells showed lytic capabilities because a notable fraction of the neuroblastoma cells were killed in the co-cultures (Fig. 1C). Interestingly, neuroblastoma cells that were not readily killed by NK cells showed enhanced membrane expression of MHC I. Of note, addition of naive NK cells, or B cells, did not result in cell lysis (Fig. 1C), in accordance with earlier work [16, 19].

These results indicate that peptide/MHC I surface expression on neuroblastoma tumors can be induced by exposure to activated but not naive NK cells. We therefore next addressed whether elevated MHC I levels might elicit increased immune recognition by CTLs.

### PRAME is an immunogenic antigen for neuroblastoma

**Figure 2:**
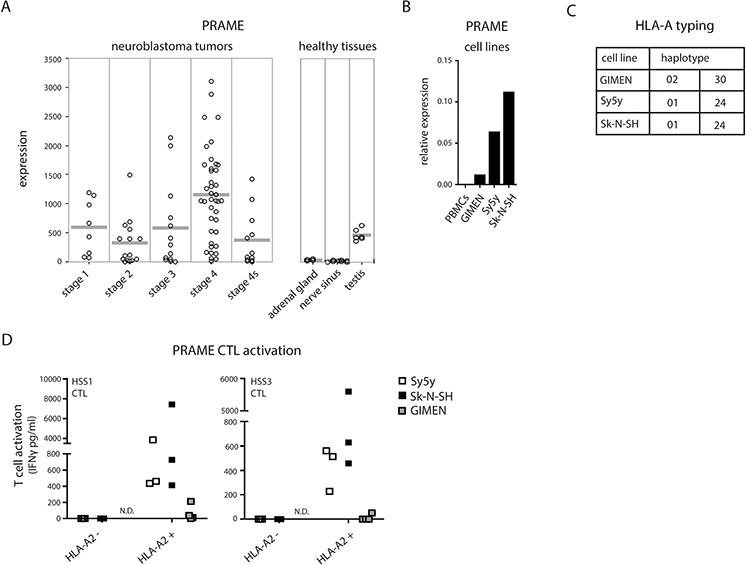
PRAME CTL recognition of neuroblastoma cells **A.** *PRAME* gene expression of 88 individual primary neuroblastoma tumors of different disease stages and healthy tissues (*NCBI GEO Accession No. GSE 16476;* http://www.ncbi.nlm.nih.gov/geo/). **B.** *PRAME* gene expression determined by qPCR of PBMCs (negative control) and neuroblastoma cell lines GIMEN, Sy5y and Sk-N-SH relative to GAPDH. **C.** Overview of HLA-A haplotypes carried by GIMEN, Sy5y and Sk-N-SH cells. **D.** Activation of PRAME_SLLQHLIGL_/A2-specific CTLs, clone HSS1 and HSS3, by HLA-A2 negative or HLA-A2 positive neuroblastoma cells.

The activation of CTLs requires triggering of their antigen-restricted T-cell receptor (TCR) by specific peptide/MHC I complexes. We performed an *in silico* data search for neuroblastoma-specific antigen expression. In an independent dataset of 88 individual neuroblastoma tumors (*NCBI GEO Accession No. GSE 16476;* http://www.ncbi.nlm.nih.gov/geo/), we found *PRAME* (also known as MAPE) to be significantly expressed in high-risk neuroblastoma tissues (Fig. 2A). Healthy neuronal tissues were negative overall for *PRAME* expression with the exception of healthy testis, hence its designation as a cancer/testis antigen [23, 24].

We first confirmed *PRAME* mRNA expression in neuroblastoma cell lines, using quantitative real-time PCR (Fig. 2B). All three neuroblastoma cell lines showed a positive signal for *PRAME* expression, though with variety between the cell lines, while *PRAME* was not detected in the negative control PBMCs. In order to address the possibility that increased MHC I surface expression may trigger CTL activation, we employed two different high affinity clones of PRAME-specific CTLs (HSS1 and HSS3). These CTL clones were isolated from patients with a mismatch bone marrow transplantation and previously described to specifically recognize PRAME-derived peptide SLLQHLIGL in combination with HLA-A2 subtype of the MHC I family [25]. Gene-profiling of the neuroblastoma cell lines showed GIMEN to carry the HLA-A2 allele whereas Sy5y and Sk-N-SH did not (Fig. 2C). As expected, neither of the HLA-A2-negative cell lines was recognized by PRAME_SLLQHLIGL_/A2-specific CTLs (Fig. 2D). However, high HLA-A2 expression attained by retroviral introduction of the HLA-A2 gene into Sy5y and Sk-N-SH cells yielded specific recognition by PRAME_SLLQHLIGL_/A2-specific CTLs (Fig. 2D; white and black squares, respectively). HLA-A2^+^ neuroblastoma cells were not recognized by A2-restricted CTLs with different antigen-specificity (minor antigen HA1, a non-neuroblastoma antigen), indicating that CTL activation was driven by antigen presentation and not a non-specific stimulation caused by lentiviral transduction (unpublished data). This data indicates that neuroblastoma cells are intrinsically capable of presenting PRAME_SLLQHLIGL_/A2 complexes and suggests that the surface display of MHC I complexes that carry immunodominant peptides is actively suppressed. In support, PRAME CTLs were unable to recognize the endogenous HLA-A2-positive GIMEN cells (Fig. 2D; grey squares). Without intervention, endogenous MHC I levels appear be too low to stimulate PRAME_SLLQHLIGL_/A2-specific CTLs whereby neuroblastoma escapes CTL-mediated anti-tumor attack.

### Activated NK cells transform neuroblastoma cells into CTL targets

**Figure 3:**
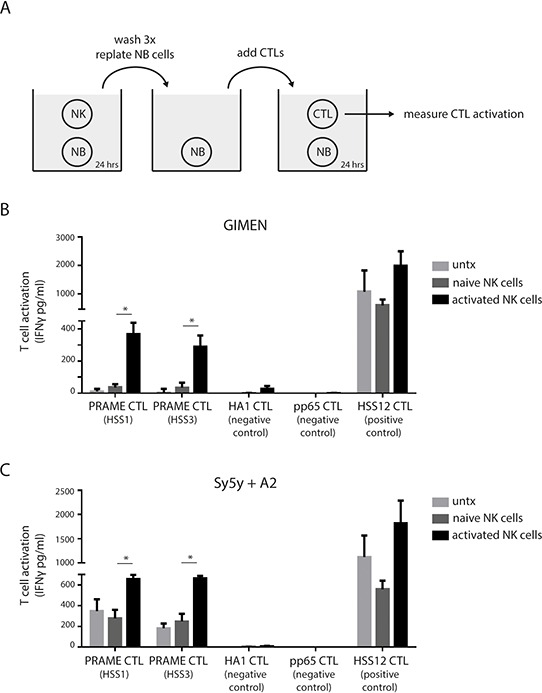
NK cells transform neuroblastoma cells into PRAME CTL targets **A.** Schematic overview of performed experiment. Neuroblastoma cells are exposed to naive or activated NK cells for 24 hours (ratio 1:1), washed thoroughly and replated in the presence of indicated CTLs. After 24 hours the culture supernatants were collected and IFNγ levels were determined by ELISA as measure of CTL activation. **B** and **C.** Activation of indicated CTLs by GIMEN cells (B) or Sy5y+A2 cells (C) left untreated or after exposure to naive or activated NK cells (*n* = 4).

We next studied whether the increase in MHC I surface display, as accomplished by prior NK cell exposure, would increase the tumor antigen-specific recognition of neuroblastoma by PRAME-specific T-cells. In a multi-step co-culture setup (Fig. 3A) GIMEN cells or HLA-A2-transduced Sy5y cells (Sy5y+A2) were exposed 1:1 to activated NK cells for 24 hours (see Fig. S1). Then either GIMEN or Sy5y+A2 cultures were washed thoroughly and replated in the presence of PRAME_SLLQHLIGL_/A2-restricted CTLs for 24 hours (30,000 neuroblastoma cells with 6,000 T-cells). GIMEN neuroblastoma cells that were modulated by activated NK cells, in contrast to naive NK cells, were recognized by PRAME_SLLQHLIGL_/A2-restricted CTLs (Fig. 3B and Fig. S3). Furthermore, A2-restricted CTLs recognizing a peptide derived from minor antigen HA1 or CMV pp65 protein (negative control) could not be activated, supporting that NK cell-modulated neuroblastoma cells do not spontaneously activate CTLs. Also, CTLs were not activated by NK cells only, both before or after incubation with neuroblastoma cells (unpublished data). As positive control A2-restricted CTLs were used that recognize a peptide derived from USP11 (ubiquitin specific peptidase 11), a highly expressed housekeeping protein, which showed T-cell activation in all conditions. The Sy5y+A2 cells, by virtue of their transduced high HLA-A2 expression rate showed enhanced basal recognition, which was however further increased after exposure to activated NK cells but not naive NK cells (Fig. 3C).

Altogether, we show that interactions with activated NK cells transform neuroblastoma cells into targets for CD8^+^ T-cells. Moreover, neuroblastoma cells display antigen specificity through MHC I presentation of the PRAME-derived SLLQHLIGL antigenic peptide.

### NK cells induce MHC I upregulation in neuroblastoma through contact-dependent IFNγ production

**Figure 4:**
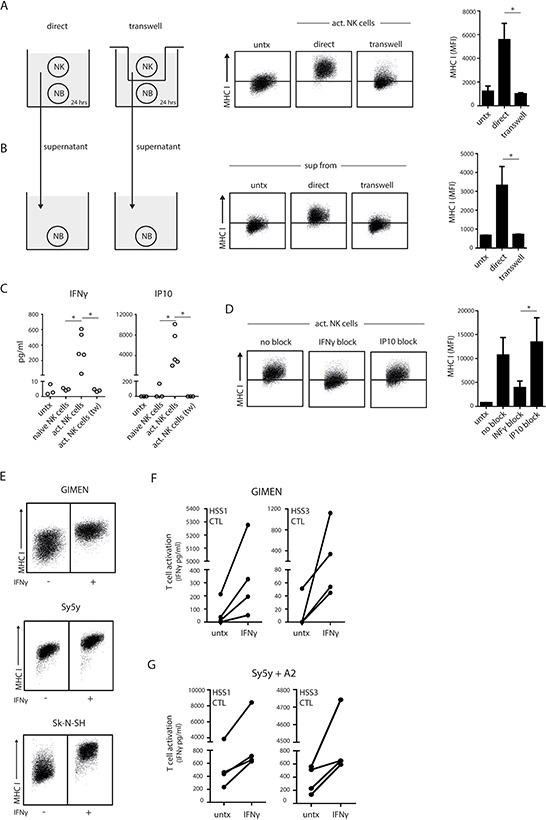
Contact-dependent release of IFNγ enhances MHC I on neuroblastoma **A.** Activated NK cells were co-cultured with neuroblastoma cells in a contact-dependent (direct) or –independent (transwell) system for 24 hrs. MHC I levels on the neuroblastoma cells were measured by flow cytometry. Representative FACS plots are shown and data was quantified in a bar graph. **B.** Supernatant of the co-cultures in A were transferred to untreated neuroblastoma cells and incubated for 24 hrs. Subsequently, MHC I levels on the neuroblastoma cells were measured by flow cytometry as in A. **C.** Concentrations of IFNγ and IP-10 were measured in the supernatant of GIMEN cells left untreated or co-cultured with naive or activated NK cells (direct and transwell). **D.** GIMEN cells were incubated with activated NK cells in the presence of IFNγ- or IP-10-blocking antibodies for 24 hours. MHC I levels were measured by flow cytometry. **E.** Neuroblastoma cells were treated with recombinant IFNγ for 24 hours and MHC I levels were measured by flow cytometry. **F** and **G.** Activation of PRAME_SLLQHLIGL_/A2-specific CTLs, clone HSS1 and HSS3, by GIMEN (F) or Sy5y+A2 (G) cells that were priorly treated with recombinant IFNγ for 24 hours.

To gain more insight into the manner by which active NK cells accomplished upregulation of antigenic peptide/MHC I complexes on neuroblastoma cells we performed various co-culture experiments. Using a transwell system, we first showed that direct contact between the NK cell and the neuroblastoma cells is necessary for MHC I upregulation, as activated NK cells are unable to do so when cell-cell contact is prevented (Fig. 4A). We collected culture supernatants of these co-cultures, transferred them to untreated neuroblastoma cells and measured MHC I changes. Only supernatants of NK-neuroblastoma co-cultures that had been allowed continuous cell-cell contact could recapitulate MHC I upregulation in fresh neuroblastoma cells (Fig. 4B). Thus, NK cells elicit class I MHC upregulation in a strict contact-dependent manner.

We hypothesized that secretion of IFNγ, which can be locally produced by activated immune cells and stimulates antigen presentation in neuroblastoma cells [26], may trigger the enhanced MHC I expression triggered by activated NK cell contact. Indeed, both IFNγ and IP10 (Interferon-induced protein 10) were present in supernatants of activated NK-neuroblastoma cell co-cultures but absent in culture supernatants of neuroblastoma cells co-cultured with B cells or naive NK cells (Fig. 4C). Moreover, there was no IFNγ production in a contact-independent co-culture using activated NK cells (Fig. 4C). Thus, cell-cell interactions between activated NK cells and neuroblastoma cells elicits the production of IFNγ. To confirm that IFNγ was required for the MHC I upregulation in neuroblastoma cells we used IFNγ blocking antibodies during the co-culture. The IFNγ blocking antibodies were able to inhibit MHC I upregulation by 60% (Fig. 4D and Fig. S4) indicating that IFNγ is the major contributor to MHC I upregulation in this co-culture. In contrast, IP10 does not appear to play a role in MHC I upregulation, as incubation with IP10 blocking antibodies did not inhibit MHC I expression levels on neuroblastoma (Fig. 4D). Additionally, we investigated the direct effect of IFNγ on neuroblastoma MHC I levels. Incubation with recombinant IFNγ could enhance MHC I surface expression on neuroblastoma cells (Fig. 4E) and subsequently increased PRAME_SLLQHLIGL_/A2-restricted CTL recognition of both GIMEN (Fig. 4F) and Sy5y+A2 cells (Fig. 4G).

In summary, contact-dependent production of IFNγ stimulates the presentation of peptide/MHC I complexes at the cell surface of neuroblastoma cells, for antigen-specific presentation and recognition by PRAME_SLLQHLIGL_/A2-restricted CTLs.

### Primary stage 4 neuroblastoma cells increase MHC I upon IFNγ stimulation

**Figure 5:**
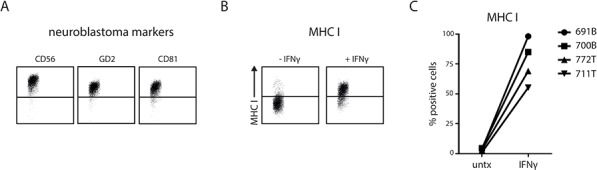
Patient-derived stage 4 neuroblastoma cells show IFNγ-induced MHC I upregulation Tumor cells were stained for neuroblastoma markers CD56, GD2 and CD81 and analyzed by flow cytometry. Representative plots are shown in **A.** MHC I surface levels were measured on indicated neuroblastoma cells (711T and 772T from tumor mass; 700B and 691B from bone marrow metastasis) which were left untreated or were stimulated with IFNγ for 24 hours. Representative plots are shown in **B.** Summarized data for all four primary neuroblastoma cells in **C.**

Finally, we asked whether primary patient-derived neuroblastoma cells do upregulate MHC I upon IFNγ exposure, as this data would validate our findings in neuroblastoma cell lines. To this end we used tumor initiating cells (TIC) that were recently obtained from primary neuroblastoma tumors or bone marrow infiltrates of stage 4 neuroblastoma patients. TICs were shown to maintain genetic and morphological phenotype of the originating tumor [27]. By flow cytometry we confirmed the neuroblastoma phenotype of four TICs as they showed positive membrane expression of CD56, GD2, and CD81 [28–30] (Fig. 5A). Steady-state MHC I levels were low, as expected for neuroblastoma tumor cells (Fig. 5B and 5C). After stimulation with IFNγ for 24 hours the surface expression of MHC I increased significantly in all four primary tumor cell types. In untreated conditions merely 3.1% (1.1–4.5) of the cells showed positive expression for MHC I which was increased up to approximately 76.9% (55.3–98.2) after IFNγ stimulation (Fig. 5C).

Thus, we confirmed in primary neuroblastoma cells the ability to upregulate MHC I surface levels in response to IFNγ. Altogether, these results indicate that the cells of primary neuroblastoma tumors actively use plasticity in immunogenic surface display of MHC I to avoid immune recognition.

## DISCUSSION

High-risk neuroblastoma is currently being treated by radiotherapy, chemotherapy, surgical resection and stem cell transplantation. More recently, these treatment strategies were complemented by antibody-based immunotherapy directed against neuroblastoma membrane marker GD2. The initial promising survival benefits of anti-GD2 therapy [6] encourages the possibility of immunotherapy in neuroblastoma. Unfortunately, the treatment was not sufficient since relapse still occurred in the majority of the patients. This suggests that immunotherapeutic approaches that induce adaptive immunity are additionally needed for complete eradication of relapsing tumor cells.

Cellular immunotherapy is still in its infancy, partly because of immune evasion: how can a cellular immunotherapy be effective if tumor cells are non-immunogenic and tumor-associated antigens are kept hidden? We here described a preclinical study that was aimed at improving TAA peptide presentation in MHC I on neuroblastoma cells. Non-immunogenic neuroblastoma cells, both from the tumor mass and bone marrow metastasis, could be pressured into surface display of immunogenic features, that were recognized by T-cells. The immunogenic potential of neuroblastoma cells exhibits a remarkable plasticity that can be exploited for clinical application.

We show that NK cells increase neuroblastoma MHC I levels in a contact-dependent manner. To date, no definitive studies on NK cell infiltration in human neuroblastoma tissue samples were performed. Though, Facchetti *et al*. succeeded in culturing NK cells from isolated lymphocytes of bulk neuroblastoma tumors [31]. While *in vitro* these NK cells proliferated and were able to produce cytokines upon IL-2 stimulation, *in vivo* the intratumoral NK cells were presumably inactive evidenced by the absence of MHC I expression in immunohistochemical stainings of neuroblastoma tumor sections [24]. In this study we have shed light on the regulation of MHC I surface expression in neuroblastoma and the manner in which NK cells contribute to the modulation thereof.

We found IFNγ to play a major role in MHC I upregulation on neuroblastoma cells. In addition to upregulation of MHC I, NK cells might also enhance the efficiency of antigen processing as Lorenzi *et al*. have shown that IFNγ can increase the expression of peptide transporter TAP and ERAP peptidases in neuroblastoma cells [26]. One could consider IFNγ treatment of neuroblastoma patients prior to cellular immunotherapy. However, because several studies have shown that interferon treatment has severe toxic effects [32–35] there is reluctance to use IFNγ in cancer patients. As an alternative, IFNγ could be delivered to tumor cells by active immune cells such as *ex vivo* expanded NK cells.

NK cell therapy has shown to induce therapeutic effects in a stage 4 neuroblastoma mouse model [36]. Moreover, in a phase II clinical trial Kloess *et al*. infused *ex vivo* expanded allogeneic NK cells into neuroblastoma patients, but tumor cytotoxicity was only observed with high-dose (≥10^7^ cells/kg) infusions [17]. Growing sufficient numbers remains a challenge for cellular therapies. We were able to show effect with using modest numbers of NK cells suggesting that merely a couple of active NK cells at the tumor site can predispose the tumor for T-cell attack. We hypothesize that dual immune pressure reduces neuroblastoma's resources for immune evasion, leaving them little option for immune escape. We propose that these new findings may be useful for exploration towards multi-attack immunotherapy combining NK cells and T-cells.

We showed for neuroblastoma cells which are modulated by interaction with active NK cells, that the presence of endogenous levels of PRAME is sufficient for productive peptide presentation to T-cells. Morandi *et al*. used peptide-pulsed T2 cells to mimic PRAME antigen presentation and were able to stimulate patient-derived PRAME-specific CTLs, which indicates not only presence but also functionality of PRAME T-cells in the neuroblastoma patient T-cell pool [37]. Altogether, we envision these T-cells to eliminate neuroblastoma tumor cells that were made immunogenic by preceding NK cell therapy.

Thus far, there is limited experimental support for direct cooperation between NK and T-cells regarding MHC I modulation and anti-tumor immunity. In human colorectal carcinoma, infiltration of both NK cells and CD8^+^ T-cells resulted in prolonged patient survival compared to tumors without NK cells [38]. In addition, MHC I analysis of colorectal carcinoma tumors in rats that were injected with IL-2 stimulated NK cells showed enhanced MHC I staining in tumor parts closer to NK cell infiltrates [39]. Similarly, enhanced influx of IL-2 activated NK and CD8^+^ T-cells into neuroblastoma tumors increased survival in a neuroblastoma mouse model [40]. Furthermore, Mocikat *et al*. showed in a lymphoma tumor model that tumor cells that were previously not recognized by CTLs become CTL targets when injected into mice in a NK cell-dependent manner [41]. Synergy between NK cells and CD8^+^ T-cells concerning tumor eradication is commonly ascribed to increased cytolytic activity within the tumor mass, regardless of the absence of specific receptors such as MHC I. Perhaps underlying such synergy is the modulatory effect of NK cells on tumor immunogenicity that predisposes the tumor for killing by CTLs.

The current study contributes to designing the optimal immunotherapy for neuroblastoma patients. Our findings here support the development of an NK cell-based cellular immunotherapy that may serve as adjuvant therapy together with an antigen-specific immune therapy. The present study contributes to relevant insights for the development of anti-neuroblastoma immunotherapies suggesting that activation of both innate activity and antigen-specific cells may hold promise for treatment of high-risk neuroblastoma patients.

## MATERIALS AND METHODS

### Cells and reagents

Neuroblastoma cell lines GIMEN, Sk-N-SH and SH-Sy5y were obtained via the Academic Medical Center of Amsterdam and maintained in DMEM supplemented with 10% FCS (Biowest), 2 mM glutamine (Life Technologies), 50 units/ml penicillin (Life Technologies) and 50 μg/ml streptomycin (Life Technologies). Microarray analysis was performed at AMC on various cell lines (*NCBI GEO Accession No. GSE 16476;* http://www.ncbi.nlm.nih.gov/geo/) confirming neuroblastoma expression profiles, which were confirmed for selected neuroblastoma markers (*LIN28B*, *MYCN*, GD2, CD56 and CD81) by qPCR and/or FACS analysis when cells arrived at UMC Utrecht (2012). Cells were frozen in individual aliquots after less than 10 passages and tested regularly for mycoplasma contamination. Tumor initiating cells were cultured as previously described [27]. T-cell clones HSS1, HSS3 are high avidity T-cell clones specific for PRAME-derived peptide SLLQHLIGL presented in the context of HLA-A*02:01 [25]. These T-cell clones recognize HLA-A*02:01 positive targets only when loaded with the PRAME peptide or when endogenously expressing PRAME [25]. T-cell clone HSS12 is specific for peptide FTWEGLYNV derived from USP11 (ubiquitin specific peptidase 11) presented in the context of HLA-A*02:01 [25]. The minor histocompatibility antigen specific T-cell clone HA1-CTL, and the cytomegalovirus specific T-cell clone pp65-CTL both also recognize their specific peptide in the context of HLA-A *02:01 and were used as negative controls. T-cell clones were stimulated every 2 weeks with 1 × 10^6^ cells/ml irradiated allogeneic PBMCs (30 Gy), 800 ng/ml PHA, and 100 IU/ml IL-2 (Chiron, Amsterdam, The Netherlands). 10–14 days after restimulation the T-cells were used for the different assays.

### Flow cytometry

For staining, cells were first detached with trypsin (Life Technologie) and washed twice in PBS containing 2% FCS (Biowest) and 0.1% sodium azide (NaN3, Sigma-Aldrich). Next, antigen nonspecific binding was prevented by prior incubation of cells with 10% mouse serum (Fitzgerald). Cells were next incubated with combinations of pacific blue, fluorescein isothiocyanate (FITC), and allophycocyanin (APC) –conjugated mouse anti–human antibodies. The following monoclonal antibodies were used: mouse-anti-human CD3 (Clone UCHT1, Biolegend), mouse-anti-human CD19 (Clone HIB19, BD Biosciences), mouse-anti-human CD56 (Clone B159, BD Biosciences), and mouse-anti-human HLA-ABC (Clone W6/32, Biolegend). Cells were acquired on FACSCanto II and analyzed using FACS Diva Version 6.13 (BD Bioscience) or FlowJo version 7.6.5 software. Data were analyzed using GraphPad Prism 5.

### Co-cultures

Peripheral blood mononuclear cells (PBMCs) from healthy donors were separated from peripheral blood by ficoll isopaque density gradient centrifugation (GE Healthcare Bio-Sciences AB) and stained with anti-CD3, anti-CD19 and anti-CD56 antibodies. CD19+ B cells and CD3-CD56+ NK cells were isolated by FACS sorting and subsequently cultured in RPMI supplemented with 10% human serum (Sigma). For NK cell activation 1000 U/ml interleukin-2 (Proleukin) and 50 ng/ml interleukin-15 (Immunotools) were added for 18 hrs. B cells, naive NK cells or activated NK cells were harvested, washed, counted and added to neuroblastoma cells at indicated effector:target ratios for 24 hrs. For cell-cell contact experiments neuroblastoma cells were added to the lower part of the transwell system (Greiner Bio-one, 1 um pore size) and immune cells were added to the upper part and cultured for 24 hrs. For blocking experiments the NK cells were mixed with anti-IFNγ (BD Biosciences) or anti-IP10 (R&D) neutralizing antibodies (1 ug/ml) and subsequently added to the neuroblastoma cells.

### Quantative real-time PCR

Total RNA was isolated using tripure (Roche) according to the manufacturer's instructions. cDNA was synthesized from up to 1 μg of total RNA using the iScript cDNA synthesis kit (Biorad). Real-time PCR was performed using IQ SYBR Green PCR Supermix (Biorad) and the CFX96 Touch Real-Time PCR Detection System (Bio-Rad), according to the manufacturer's instructions. PCR assays were done in triplicate. Data were calculated as values relative to GAPDH and further analyzed using Graphpad Prism 5.

### HLA typing

One million cells were used for typing of the HLA-A locus of neuroblastoma cells. The LABType SSO HLA A Locus typing kit (OneLambda) was used according to manufacturer's instructions.

### Retroviral transduction

Neuroblastoma cells were transduced with a retroviral vector encoding for HLA-A*0201 and the marker gene NGF-R. By MACS separation the NGF-R positive neuroblastoma cells were enriched.

### CTL activation

Neuroblastoma cells were pretreated with NK cells (1:1) or 1000 U/ml recombinant IFNγ (eBioscience) as indicated for 24 hrs. Then, neuroblastoma cells were harvested, washed thoroughly and replated in the presence of CTLs (30,000 neuroblastoma cells with 6,000 CTLs). After 24 hrs culture supernatants were collected and CTL activation was measured as production of IFNγ, IL-2 and TNF.

### Cytokine detection

Cytokine concentrations were measured by the MultiPlex Core Facility of the LTI using Luminex technology with in house developed bead-sets and Bio-Plex Manager Version 6.1 software (Bio-Rad Laboratories) as previously described [42]. IFNγ concentrations were measured using ELISA (eBiosciences).

### Statistics

Differences in MHC I surface expression levels, T cell activation levels, and cytokines (IFNγ and IP10) secretion levels were analyzed with non-parametric Mann-Whitney *U* test between separate groups. Values of *p* < 0.05 were considered significant. Analyses were performed with GraphPad Prism 5 software.

## SUPPLEMENTARY MATERIALS FIGURES


