# Supplementary material for: Natural killer cells facilitate PRAME-specific T-cell reactivity against neuroblastoma
Source: Oncotarget. 2015 Oct 6;6(34):35770–81. doi: 10.18632/oncotarget.5657 (PMC4742140; doi:10.18632/oncotarget.5657)
Supplement: Supplementary file 1 [file oncotarget-06-35770-s001.pdf]

SUPPLEMENTARY FIGURES

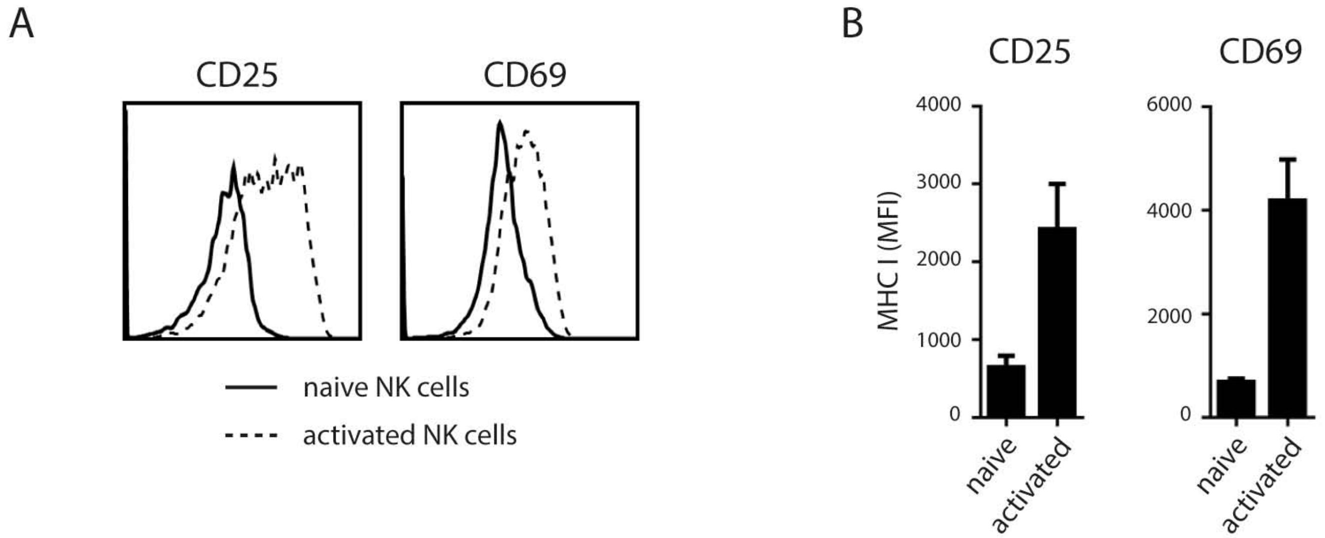

Supplementary Figure S1: NK cell activation. CD3^{-}CD56^{+} NK cells were FACS sorted from healthy donor PBMCs and cultured in absence or presence of IL2 and IL15 for 18 hours. Membrane expression of CD25 and CD69 was determined on the NK cells by flow cytometry as measure of cell activation. Representative FACS plots are shown in **A.** Data was quantified in **B.**

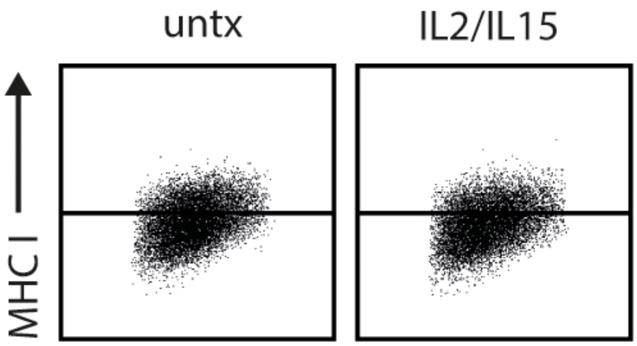

Supplementary Figure S2: IL2/IL15 cytokines do not influence neuroblastoma MHC I levels. GIMEN cells were left untreated or stimulated with IL2 and IL15 for 24 hours. MHC I levels were measured by flow cytometry.

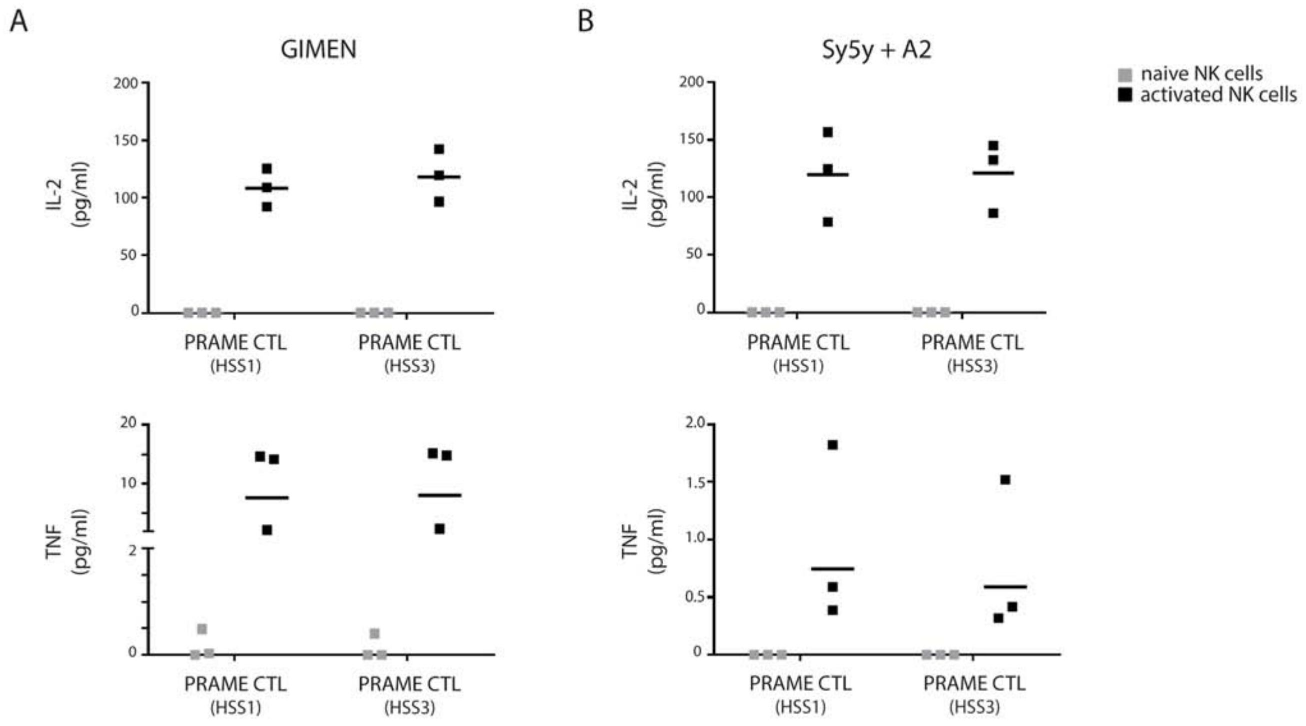

Supplementary Figure S3: PRAME-CTL activation by NK cell-modulated neuroblastoma cells. GIMEN **A.** and Sy5y+A2 **B.** cells were exposed to naive or activated NK cells, derived from three different donors, for 24 hours (ratio 1:1), washed thoroughly and replated in the presence of PRAME-specific CTLs. After 24 hours the culture supernatants were collected. IL-2 and TNF levels were determined by Luminex as measure of CTL activation.

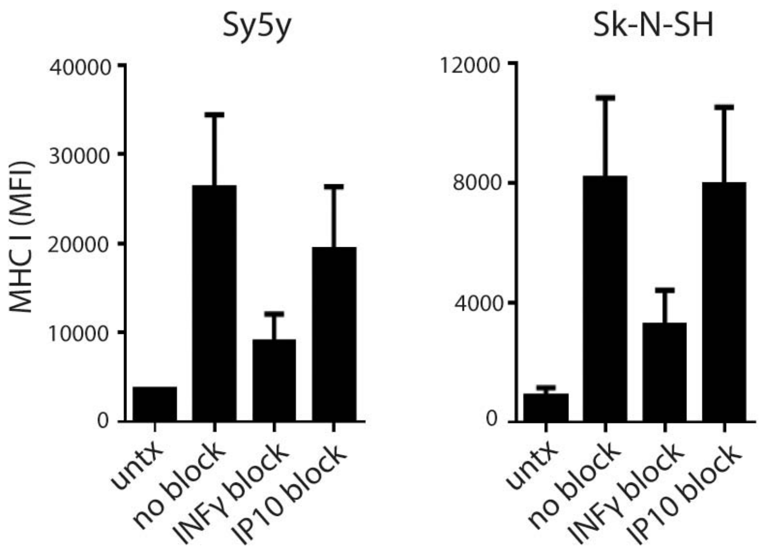

Supplementary Figure S4: NK cell-induced MHC I upregulation on neuroblastoma is IFNγ-dependent. Sy5y and Sk-N-SH cells were incubated with activated NK cells in the presence of IFNγ- or IP-10-blocking antibodies for 24 hours. MHC I levels on the neuroblastoma cells were measured by flow cytometry.
